# Editorial: Lipids and membrane contacts – structure, functional aspects and implications on ageing, cell death and autophagy, volume II

**DOI:** 10.3389/fcell.2025.1589044

**Published:** 2025-03-19

**Authors:** Christopher T. Beh, Alexandre Toulmay, Patrick Rockenfeller

**Affiliations:** ^1^ Department of Molecular Biology and Biochemistry, Simon Fraser University, Burnaby, BC, Canada; ^2^ Cell Biology, University of Texas Southwestern Medical Center, Dallas, TX, United States; ^3^ Chair of Biochemistry and Molecular Medicine, Center for Biomedical Education and Research (ZBAF), University of Witten/Herdecke, Witten, Germany

**Keywords:** membrane contact sites (MCSs), lipid exchange and transport, autophagy, membrane homoeostasis, membrane signaling, membrane-related diseases

Given the surge of developments related to membrane contact sites (MCSs), the first volume of the Research Topic “Lipids and membrane contacts in yeast–structure, functional aspects and implications on aging, cell death and autophagy” required revisiting. Building on the first volume, the second volume includes papers that explore intricacies of lipid droplet contact with the endoplasmic reticulum (ER), as well as mitochondrial crosstalk with the ER and lysosomes during mitophagy and disease progression, including carcinogenesis. This next installment also includes a timely article reviewing the experimental challenges of studying MCSs and their tether subunits, alongside new and adapted methods for MCS identification and analysis.

MCSs act as membrane connectors, establishing intracellular highways for lipid trafficking while also functioning as hubs for inter-compartment communication and coordinating membrane stress responses (Rockenfeller and Gourlay, 2018; Prinz et al., 2020; Zaman et al., 2020). They also play multiple roles in regulating metazoan calcium signaling (Ke et al., 2025; Stefan, 2020). Less appreciated, however, MCSs are crucial for the *de novo* biogenesis of some membrane compartments, such as autophagosomes, which depend on membrane contacts with the ER and mitochondria for assembly (Herrera-Cruz and Simmen, 2017; Metur and Klionsky, 2020; Molino et al., 2017; Gómez-Sánchez et al., 2018; Valverde et al., 2019; Zwilling and Reggiori, 2022). Beyond autophagosome biogenesis, autophagy-related MCSs direct selective organelle degradation, including mitochondrial autophagy or mitophagy (Kohler et al., 2020; Zwilling and Reggiori, 2022).

MCSs regulate the balance of mitochondrial assembly and, through mitophagy, remove damaged mitochondria to maintain a stable population that meets cellular demands (Schrader et al., 2015). MERCs (Mitochondria–ER contact sites) initiate mitophagy for the selective degradation of damaged mitochondria (Yang et al., 2020). As a MERC-interacting protein, FUNDC1 (FUN14 Domain Containing 1) is a key regulator of mitochondrial engulfment during mitophagy. FUNDC1 is an integral mitochondrial outer-membrane protein containing a specific domain for interaction with LC3, the mammalian homologue of Atg8 that regulates autophagosome formation (Liu et al., 2012; Lee et al., 2025). The dynamics of this interaction is particularly important in hypoxic cells where FUNDC1 accumulates at MERCs and then binds LC3 to recruit autophagosomes and initiate mitophagy (Liu et al., 2012; Wu et al., 2016). The role of FUNDC1 in mediating hypoxia induced mitochondrial degradation has been established in heart injury, and it is a promising target for treating tumours and other human disorders (Zhang et al., 2016; Tan et al., 2022; Atici et al., 2023; Dong and Zhang, 2024). In this context, Li et al. contribute to this Research Topic with their review article “Multiple roles of mitochondrial autophagy receptor FUNDC1 in mitochondrial events and kidney disease,” focusing on the role of FUNDC1 in renal disease. The authors explore the mechanisms by which FUNDC1 regulates mitophagy in the context of diseases that affect the kidney, an important metabolic organ.

Kumar et al. contribute to this Research Topic with a comprehensive review, “The evolving landscape of ER-LD contact sites,” in which they explore lipid droplet (LD) contacts with the ER. The authors describe the unique contacts that bridge the LD monolayer with the ER bilayer and discuss their role in LD biogenesis within the ER, as well as in maintaining cytoplasmic LDs and ER/lipid homeostasis. They highlight the importance of Seipin, the mammalian homologue of yeast Sei1/Fld1, in defining sites of LD biogenesis occurs in ER and in establishing ER-LD contacts for LD maintenance. The review also covers current insights into molecular tethers forming ER-LD interfaces, and this study complements articles in the first volume of this Research Topic, which detailed LD contacts with several different membrane compartments.

In “Imaging and proteomics toolkits for studying organelle contact sites” Gamuyao and Chang present technical advances for analyzing tethering complexes, further expanding the scope of this Research Topic of articles and reviews. Beyond genetic approaches for the functional analysis of MCSs, this work focuses on imaging techniques using MCS reporters and proximity labeling to profile membrane tethering complexes. It also discusses the challenges of imaging MCSs and the unbiased identification of associated components.

The review article “The role of extended synaptotagmin at membrane contact sites in cancer research” by Pan et al. explores how MCSs influence cancer progression and discusses how changes in MCSs might be utilized as a diagnostic biomarker for cancer research. The authors focus on the role of extended synaptotagmins (E-Syts) in calcium and lipid signaling, linking them to tumor proliferation, progression, metastasis, apoptosis, drug resistance, and treatment. By highlighting the unique role of E-Syts at MCSs in integrating both calcium and lipid signaling pathways, the review uses E-Syts as a starting point to describe a different direction for understanding cancer.

This second Research Topic of articles addresses some of the original questions we raised about challenges confronting MCS research (Rockenfeller et al., 2022). However, questions remain regarding how MCSs regulate metabolic pathways and their contribution to disease pathology, and these will continue to drive research as new MCSs are discovered. With the identification of additional tethering complexes, future research will likely focus on MCS dynamics in response to changes in metabolic, developmental, and stress programs. Perhaps these studies will lay the groundwork for yet another future Research Topic on MCS regulation.
